# C/EBP transcription factors regulate NADPH oxidase in human aortic smooth muscle cells

**DOI:** 10.1111/jcmm.12289

**Published:** 2014-05-06

**Authors:** Simona-Adriana Manea, Andra Todirita, Monica Raicu, Adrian Manea

**Affiliations:** Molecular and Cellular Pharmacology – Functional Genomics Laboratory, Institute of Cellular Biology and Pathology “Nicolae Simionescu” of the Romanian AcademyBucharest, Romania

**Keywords:** NADPH oxidase, C/EBP, atherosclerosis, oxidative stress, inflammation

## Abstract

In atherosclerosis, oxidative stress-induced vascular smooth muscle cells (SMCs) dysfunction is partially mediated by up-regulated NADPH oxidase (Nox); the mechanisms of enzyme regulation are not entirely defined. CCAAT/enhancer-binding proteins (C/EBP) regulate cellular proliferation and differentiation, and the expression of many inflammatory and immune genes. We aimed at elucidating the role of C/EBP in the regulation of Nox in SMCs exposed to pro-inflammatory conditions. Human aortic SMCs were treated with interferon-γ (IFN-γ) for up to 24 hrs. Lucigenin-enhanced chemiluminescence, real-time PCR, Western blot, promoter-luciferase reporter analysis and chromatin immunoprecipitation assays were employed to investigate Nox regulation. IFN-γ dose-dependently induced Nox activity and expression, nuclear translocation and up-regulation of C/EBPα, C/EBPβ and C/EBPδ protein expression levels. Silencing of C/EBPα, C/EBPβ or C/EBPδ reduced significantly but differentially the IFN-γ-induced up-regulation of Nox activity, gene and protein expression. *In silico* analysis indicated the existence of typical C/EBP sites within Nox1, Nox4 and Nox5 promoters. Transient overexpression of C/EBPα, C/EBPβ or C/EBPδ enhanced the luciferase level directed by the promoters of the Nox subtypes. Chromatin immunoprecipitation demonstrated the physical interaction of C/EBPα, C/EBPβ and C/EBPδ proteins with the Nox1/4/5 promoters. C/EBP transcription factors are important regulators of Nox enzymes in IFN-γ-exposed SMCs. Activation of C/EBP may induce excessive Nox-derived reactive oxygen species formation, further contributing to SMCs dysfunction and atherosclerotic plaque development. Pharmacological targeting of C/EBP-related signalling pathways may be used to counteract the adverse effects of oxidative stress.

## Introduction

Compelling evidence indicates that the reactive oxygen species (ROS) produced by members of the NADPH oxidase (Nox) family are important regulators of key biological activities such as cell growth, proliferation, differentiation, migration and apoptosis [1–3]. Produced in excess, Nox-derived ROS are highly detrimental in numerous cardiometabolic pathologies including atherosclerosis, diabetes, hypertension and obesity [4–6]. The uncover of the stream of signalling molecules responsible for Nox up-regulation and the ensuing ROS production may be used to define strategies to counteract the adverse effects of oxidative stress.

Nox up-regulation correlated with atherosclerotic lesion progression was demonstrated in various animal models and in humans [7–10]; their specific role is yet to be discovered. Oxidative stress has been linked to the dysfunction of vascular smooth muscle cells (SMCs), which further promotes and accelerates the development of atherosclerotic lesions. Phenotypic alterations of SMCs represent a maladaptive response to vascular insults and are considered to be partially mediated by Nox-derived ROS [11,12]. Smooth muscle cells express typically Nox1, Nox4 and Nox5 subtypes that are differentially distributed within the cellular compartments and control important redox-sensitive signalling pathways [13].

In previous studies, we have demonstrated that the Nox activity and expression are up-regulated by interferon γ (IFN-γ) in human aortic SMCs by direct and indirect mechanisms involving signal transducer and activator of transcription (STAT), nuclear factor-kB (NF-kB) and activator protein-1 (AP-1) transcription factors [14,15]. In addition to the archetypal Jak (Janus-activated kinase)/STAT pathway, IFN-γ also activates non-STAT signalling pathways including the basic-leucine zipper (bZip) transcription factor family, CCAAT/enhancer-binding proteins (C/EBP) [16]. Thus far, the implication of C/EBP in the regulation of Nox was not demonstrated.

C/EBP family comprises six members (C/EBP-α, -β, -δ, -γ, -ε, -ξ), each with a distinct cell and tissue distribution. In hepatocytes, adipocytes and haematopoietic cells, various C/EBP subtypes are critical regulators of many biological activities including cellular proliferation and differentiation, immune and inflammatory responses. Still, the precise function and regulation of C/EBP in the resident vascular cells is not known. Upon activation, C/EBP are able to form homo- or heterodimers with each other or heterodimers with other transcription factors and interact with the CCAAT box motif in the enhancers and promoters of target genes. In addition, C/EBP can shape the chromatin conformation by recruiting various transcriptional co-factors [17].

Among the C/EBP family, the activity and expression of three members (C/EBP-α, -β and -δ) have been shown to be regulated by a number of inflammatory agents leading to enhanced expression of many cytokines, chemokines, acute phase proteins and immunoglobulins [18–20]. As inflammation and oxidative stress are interrelated in atherogenesis, in this study, we aimed at investigating the implication of C/EBP-α, -β and -δ transcription factors in regulation of Nox in cultured human aortic SMCs exposed to pro-inflammatory conditions. On the basis of the fact that cytokine milieu present in atherosclerosis promotes Nox expression, in this study, we have used IFN-γ, a potent pro-inflammatory cytokine secreted in the atheroma artery-infiltrating T cells. The data provide evidence that C/EBP-α, -β and -δ play a role in mediating IFN-γ-induced Nox expression and function in SMCs and highlights the potential involvement of the C/EBP family in transducing the effects of pro-inflammatory stimuli *via* redox-sensitive signalling pathways in atherosclerosis.

## Materials and methods

### Materials

Standard chemicals, antibodies, siRNA, reagents and molecular biology kits were obtained from Sigma-Aldrich (Schnelldorf, Germany), Santa Cruz Biotechnology (Dallas, TX, USA), Invitrogen (Vienna, Austria), and Qiagen (Düsseldorf, Germany). The C/EBP-α, -β and -δ expression vectors were from Thermo/OpenBiosystems (Huntsville, AL, USA). The sequences of the oligonucleotide primers used to amplify various regions of Nox1, Nox4 and Nox5 gene promoters are depicted in the Table S1. The primers used in the chromatin immunoprecipitation (ChIP) assays to amplify DNA fragments derived from the promoters of human p21 and c-Myc genes were from R&D Systems (Vienna, Austria).

### Cell culture

Previously characterized human aortic SMCs were used [21]. The cell isolation was done in accordance with the institutional ethical guidelines. Confluent quiescent cells (at passage 7–10) cultured for 24 hrs in serum-free DMEM (5.5 mM glucose) were further exposed for up to 24 hrs to IFN-γ (5–100 ng/ml) in the absence or presence of specific siRNA for C/EBP-α, -β and -δ.

The study was conducted in accordance with the ethical principles for medical research involving human subjects (World Medical Association Declaration of Helsinki), and the local committee on human research approved the study protocol.

### Assessment of Nox activity and intracellular ROS production

The lucigenin-enhanced chemiluminescence assay was employed to estimate the NADPH oxidase-dependent O_2_^•−^ production in membrane fractions obtained from cultured SMCs [22,23]. Samples were equilibrated for 30 min. at 37°C in 50 mM phosphate buffer pH 7.0 containing 1 mM CaCl_2_ and protease inhibitor cocktail prior to the addition of lucigenin (5 μM) and NADPH (100 μM). The light emission was recorded every second for 15 min. in a luminometer (Berthold, Vienna, Austria). Following subtracting the blank chemiluminescence signal, the total Nox activity was calculated from the ratio of mean light units to total protein level and expressed as arbitrary units.

Dichlorofluorescein (DCF) assay was used to determine ROS production in intact cells as described previously [24]. Briefly, cultured human aortic SMCs were loaded with 5 μM 5(6)-carboxy-2′,7′-DCF diacetate for 30 min. in the dark at 37°C, detached and resuspended in modified Hepes-buffered saline solution, comprising (in mmol/l): 145 NaCl, 5 KCl, 1.8 CaCl_2_, 1 MgCl_2_, 1 Na_2_HPO_4_, 5 glucose, 25 Hepes (pH 7.4). The cells were dispersed at 10^4^/well into a 96-well microplate reader (Tecan, Grödig, Austria) and the DCF fluorescence emission was detected at 585 nm with an excitatory wavelength of 485 nm. The ROS production was calculated from the ratio of relative fluorescence units to total protein level and expressed as arbitrary units.

### Cell impedance measurements

To evaluate the effects of IFN-γ on SMCs dynamics, the impedance-based assay for real-time monitoring of cell dynamics (xCELLigence, Roche, Bucharest, Romania) was employed. The cells were seeded at 5 × 10^3^/well in 16-well E-Plates™ and 24 hrs later were exposed to 5–100 ng/ml of IFN-γ. The results were analysed using RTCA™ software.

### MTT cell proliferation assay

To investigate the implication of C/EBP transcription factor family in the modulation of SMCs proliferation, Vybrant™ MTT cell proliferation assay was used according to the manufacturer's protocol (Molecular Probes, Vienna, Austria).

### Real-Time PCR

Total cellular RNA was isolated from cultured SMCs using an RNA kit (Sigma-Aldrich). The mRNA levels were quantified by amplification of cDNA using a real-time thermocycler (LightCycler®480 II; Roche) and SYBR™ Green I chemistry. Oligonucleotide primers were as follows: Nox1 (NM_013955) sense: 5′-CACAAGAAAAATCCTTGGGTCAA-3′, and antisense: 5′-GACAGCAGATTGCGACACACA-3′; Nox4 (NM_016931) sense: 5′-TGGCTGCCCATC TGGTGAATG-3′, and antisense: 5′-CAGCAGCCCTCCTGAAACATGC-3′; Nox5 (NM_024505) sense: 5′-CAGGCACCAGAAAAGAAAGCAT-3′, and antisense: 5′-ATGTTGTCTTGGACACCTTCGA-3′; β-Actin (NM_001101) sense: 5′- CTGGCACCCAGCACAATG -3′, and antisense 5′- GCCGATCCACACGGAGTACT -3′. Optimized amplification conditions were 0.2 μM of each primer, 2.5 mM MgCl_2_, annealing at 60°C and extension at 72°C for 40 cycles. The gene expression levels of Nox1, Nox4 and Nox5 were normalized to β-Actin. The relative quantification was done using the comparative C_T_ method and expressed as arbitrary units [25].

### Western blot

Cell lysate preparation and Western blot analysis were done as described previously [14,15]. Briefly, cultured SMCs were washed twice in ice-cold PBS before lysis in 2× Laemmli's electrophoresis sample buffer and incubated at 95°C for 20 min. Amido Black method [26] was used to determine the protein concentration. Equal amounts of protein (50 μg) were run on 10% SDS-PAGE and electroblotted onto nitrocellulose membranes (Bio-Rad, Bucharest, Romania). The membranes were exposed to blocking reagent TBS Blotto A (sc-2333), and then incubated overnight at 4°C with the primary antibodies against Nox1 (rabbit polyclonal, sc-25545), Nox4 (goat polyclonal, sc-55142), Nox5 (goat polyclonal, sc-34707), C/EBPα (goat polyclonal, sc-9314), C/EBPβ (rabbit polyclonal, sc-150), C/EBPδ (rabbit polyclonal, sc-636), Histone H1 (rabbit polyclonal, sc-34464) or β-Actin (mouse monoclonal, sc-47778), followed by horseradish peroxidase-conjugated secondary antibodies. The protein bands were taken with a digital detection system (ImageQuant LAS 4000; Fujifilm, Tokyo, Japan). Quantification (TotalLab™, Newcastle upon Tyne, UK) of Nox1, Nox4, Nox5 and C/EBP proteins was done by normalization to β-Actin protein or Histone H1 (nuclear translocation assays) and expressed as arbitrary units.

### Transfection of siRNA

The siRNA directed to C/EBPα (sc-37047), C/EBPβ (sc-29229), C/EBPδ (sc-37722) or scrambled (C siRNA, sc-37007; 20 nM) was transfected into cultured human aortic SMCs using Hiperfect™ reagent, according to the manufacturer's protocol. Silencing efficiency was evaluated by Western blot, 48–72 hrs after siRNA transfection.

### Plasmid construction, transient transfection and luciferase assay

The proximal promoters (≈1000 bp) of the human Nox1, Nox4 and Nox5 genes were cloned into the pGL3 basic vector and characterized as previously described [14,15,27]. Transient transfection was performed as in [28] using Superfect™ reagent. The plasmid concentrations were as follows: 1 μg of promoter-luciferase construct (pNox1/4/5-Luc), 0.1 μg pSV-β-galactosidase vector, 0.3 μg of expression vector (C/EBP-α, -β and -δ) or their corresponding empty vectors controls. The DNA/Superfect ratio was 1:7.5 (wt/wt). The promoter activity was calculated from the ratio of firefly luciferase to β-galactosidase levels and expressed as arbitrary units.

### ChIP assay

Chromatin immunoprecipitation assay was performed as previously indicated [15,29]. Polyclonal antibodies against C/EBP-α (sc-9314), C/EBP-β (sc-150) and C/EBP-δ (sc-636) were used. PCR was done with primers sets (≈200 bp amplicon size) spanning the entire region of the Nox1, Nox4 and Nox5 proximal promoter regions (≈1000 bp). ‘No-antibody’, IgG and negative controls from genomic regions, which do not contain predicted elements (*e.g*. c-Myc gene promoter), were employed. A DNA fragment derived from p21 gene promoter containing C/EBP elements was immunoprecipitated and served as positive control. The specificity of PCR products was analysed by gel electrophoresis. Input DNA was amplified for each sample in parallel experiments.

### Data analysis

Data obtained from at least three independent experiments performed in duplicate were expressed as means ± SD. Statistical analysis was performed by one-way anova and Tukey's range test; *P* < 0.05 was considered statistically significant.

## Results

### Up-regulation of Nox activity and intracellular ROS production by IFN-γ

**Fig. 1:**
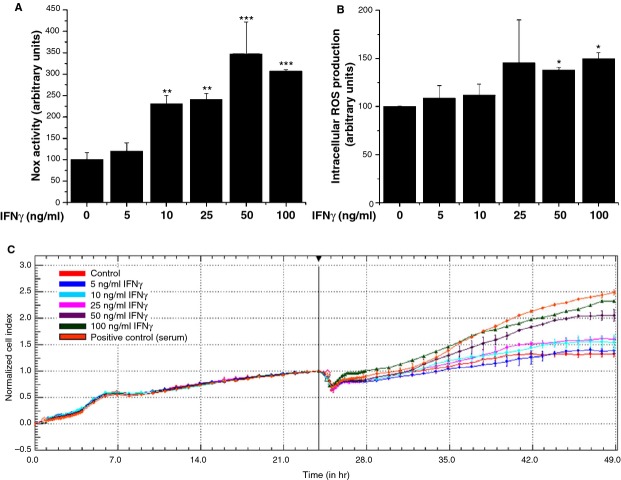
Interferon-γ (IFN-γ) induces Nox activity (**A**) and intracellular reactive oxygen species (ROS) production (**B**), and proliferation (**C**) of human aortic smooth muscle cells (SMCs). Cultured quiescent SMCs were exposed to the indicated concentrations of IFN-γ for 24 hrs. The Nox activity was detected by lucigenin-enhanced chemiluminescence and the intracellular formation of ROS by dichlorofluorescein fluorescence; *n* = 3, *P < 0.05, **P < 0.01, ***P < 0.001. *P* values were taken in relation to vehicle-exposed cells. The concentration-dependent effect of IFN-γ on SMCs dynamics was monitored continuously for 48 hrs using E-Plates™technology. Quiescent cells were considered as baseline control and the 10% serum condition was used as positive control of SMCs proliferation. Data shown are representative of three independent experiments performed in triplicate.

To evaluate the effect of IFN-γ on Nox activity, dose-dependent experiments were performed. Quiescent SMCs were exposed to 5–100 ng/ml IFN-γ for 24 hrs and the NADPH-dependent O_2_^•−^ production was determined by lucigenin-enhanced chemiluminescence. Interferon-γ induced significant increases in the Nox activity in a dose-related manner. Maximal effects were observed in the range of 20–100 ng/ml IFN-γ (≈2.5- to 3-fold increase; Fig. 1A).

To validate the lucigenin-based technique, DCF assay was used to detect ROS in intact cells. A similar pattern of IFN-γ-induced ROS production was found by using both methods. However, up-regulation of intracellular ROS production attained a significant elevation in the range of 50–100 ng/ml IFN-γ (≈1.25- to 1.45-fold increase; Fig. 1B). Therefore, in further experiments, we have used 50 ng/ml IFN-γ.

### IFN-γ induces SMCs proliferation

To determine the effect of IFN-γ on SMCs proliferation, we used an emerging technology, the impedance-based assay for real-time monitoring of cell dynamics (xCELLigence). Smooth muscle cells were exposed to various doses of IFN-γ (5–100 ng/ml) in a single step and the data were analysed using RTCA™ software. As depicted in Figure 1C, exposure to increasing concentrations of IFN-γ affected SMCs dynamics in a dose-dependent manner. To confirm the impedance-based measurements and to evaluate the role of C/EBPs in mediating SMCs proliferation, MTT assay was employed. As shown in Figure S1, IFN-γ-induced SMCs proliferation is mediated by C/EBPα-dependent mechanisms.

### IFN-γ augments Nox expression in SMCs

**Fig. 2:**
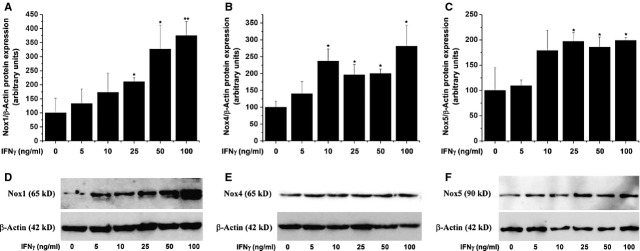
Interferon-γ (IFN-γ) up-regulates Nox1 (**A**), Nox4 (**B**) and Nox5 (**C**) protein expression levels in smooth muscle cells (SMCs). Quiescent cells were treated with increasing doses of IFN-γ (5–100 ng/ml) for 24 hrs. The quantification of Nox1, Nox4 and Nox5 protein levels was done by Western blot. Representative immunoblots depicting Nox1/4/5 protein levels following incubation of SMCs with various concentration of IFN-γ (**D**–**F**). *n* = 5, *P < 0.05, **P < 0.01. *P* values were taken in relation to vehicle-exposed cells.

Dose–response analysis was performed to evaluate whether increasing concentrations of IFN-γ affects Nox expression in human cultured SMCs. Quiescent cells were exposed to 5–100 ng/ml IFN-γ for 24 hrs and the mRNA and protein expression levels of Nox1, Nox4 and Nox5 subtypes were assessed by real-time PCR and Western blot respectively. Interferon-γ induced dose-dependent but dissimilar increases in Nox proteins expression levels. As depicted in Figure 2, IFN-γ greatly induced Nox1 protein expression. Nox4 and Nox5 protein levels were slightly but significantly up-regulated in response to IFN-γ treatment. A similar pattern of increased expression of mRNA levels of the Nox1, Nox4 and Nox5 isotypes was detected (data not shown).

### IFN-γ activates C/EBP transcription factors in SMCs

**Fig. 3:**
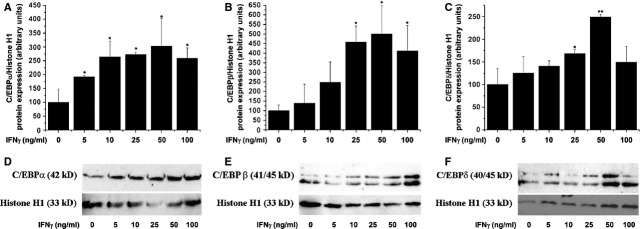
Interferon-γ (IFN-γ) activates CCAAT/enhancer-binding proteins-α (C/EBP-α) (**A**), C/EBP-β (**B**) and C/EBP-δ (**C**) in smooth muscle cells (SMCs). Cultured quiescent SMCs were exposed to the indicated concentrations of IFN-γ for 1 hr. Nuclear translocation of the C/EBP transcription factors was assessed by Western blot. Representative immunoblots depicting the C/EBP protein levels in nuclear fraction following incubation of SMCs with IFN-γ (**D**–**F**). *n* = 5, *P < 0.05, **P < 0.01. *P* values were taken in relation to vehicle-exposed cells.

To examine the involvement of C/EBP transcription factors in mediating IFN-γ signalling in SMCs, dose–response analysis was done to asses C/EBP-α, -β and -δ activation. Stimulation of SMCs with 5–100 ng/ml IFN-γ for 1 hr triggered significant but differentially nuclear translocation of C/EBP-α, -β and -δ transcription factors. A dose-dependent increase in nuclear translocation was detected in the case of C/EBP-α and -β. Stimulation of SMCs with the indicated concentrations of IFN-γ led to a gradual activation of C/EBP-δ in the range of 5–50 ng/ml IFN-γ (Fig. 3).

**Fig. 4:**
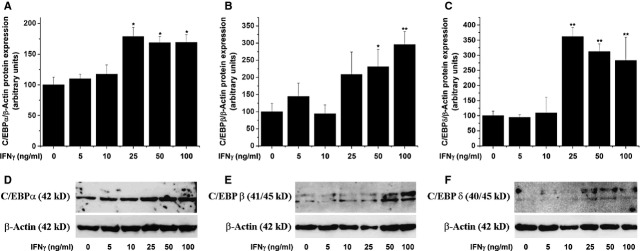
Interferon-γ (IFN-γ) up-regulates CCAAT/enhancer-binding proteins-α (C/EBP-α; **A**), C/EBP-β (**B**) and C/EBP-δ (**C**) proteins in smooth muscle cells (SMCs). Quiescent SMCs were stimulated with IFN-γ (5–100 ng/ml) for 24 hrs. The total C/EBP protein levels were assessed by Western blot. Representative immunoblots showing the C/EBP protein regulation in IFN-γ-exposed aortic SMCs (**D**–**F**); *n* = 5, *P < 0.05, **P < 0.01. *P* values were taken in relation to vehicle-exposed cells.

To investigate further the complexity of C/EBP regulation under pro-inflammatory conditions, the level of C/EBP proteins was investigated in quiescent SMCs exposed (24 hrs) to increasing concentrations of IFN-γ (5–100 ng/ml). As depicted in Figure 4, IFN-γ induced a pronounced up-regulation of the C/EBP-α, -β and -δ protein expression levels.

### C/EBP transcription factors mediate IFN-γ-induced up-regulation of Nox

**Fig. 5:**
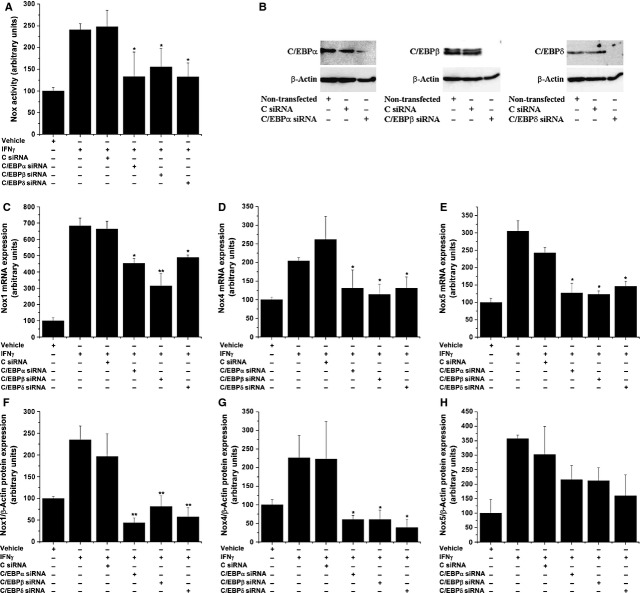
CCAAT/enhancer-binding proteins-α (C/EBP-α), C/EBP-β and C/EBP-δ mediate IFN-γ-induced up-regulation of Nox activity (**A**), mRNA (**C**–**E**) and protein (**F**–**H**) expression levels. Quiescent smooth muscle cells (SMCs) were exposed (24 hrs) to 50 ng/ml IFN-γ in the absence/presence of siRNA sequences directed to silence the expression of various C/EBP subtypes. Nox activity, gene and protein expression were assayed by lucigenin-enhanced chemiluminescence, real-time PCR and Western blot respectively. Representative immunoblots depicting the decrease in C/EBP proteins upon siRNA transfection in SMCs (**B**); *n* = 4, *P < 0.05, **P < 0.01. *P* values were taken in relation to C siRNA-transfected cells stimulated with IFN-γ.

To search for the involvement of C/EBP-associated signalling pathways in mediating IFN-γ-induced up-regulation of Nox, SMCs were transiently transfected with specific siRNA sequences directed to each of the three C/EBP isoforms or scrambled (control). To achieve a significant down-regulation of C/EBP proteins, cultured SMCs were exposed 48 hrs after siRNA transfection to 50 ng/ml IFN-γ for additional 24 hrs. The results showed that IFN-γ-induced Nox activity (≈2.5-fold) was significantly down-regulated (≈50%) by siRNA-mediated silencing of C/EBP-α, -β or -δ (Fig. 5A). To investigate whether other stimuli regulate Nox *via* a C/EBP-dependent mechanism, we used angiotensin II (AngII), a potent inducer of Nox expression and activity. As depicted in Figure S2, stimulation of SMCs with AngII induced dose-dependent increases in Nox activity. Moreover, silencing of each C/EBP isoform led to significant, but differential decreases in the up-regulated NADPH-dependent O_2_^•−^ production. Figure 5B shows the decrease in C/EBP proteins upon siRNA transfection in SMCs. Silencing of C/EBP-α, -β or -δ led to a disparate reduction in IFN-γ-augmented Nox1-, Nox4-, and Nox5 mRNA and proteins (Fig. 5C–H).

### Promoter analysis of the human Nox1, Nox4 and Nox5 genes

**Fig. 6:**
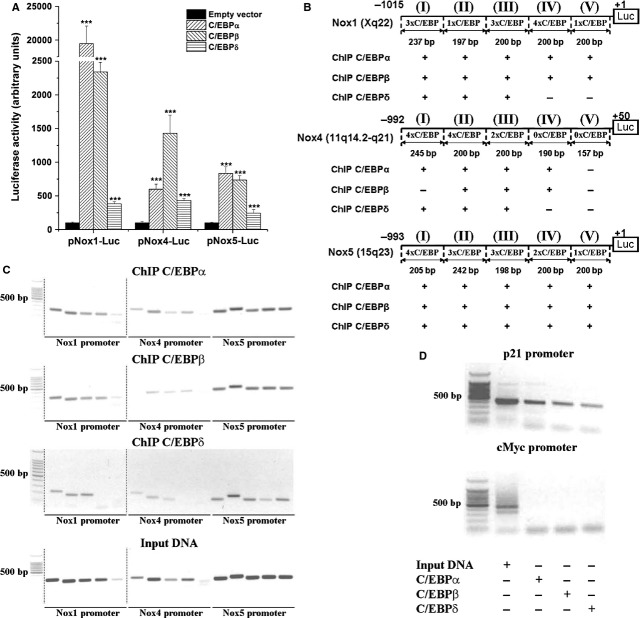
Functional analyses of CCAAT/enhancer-binding proteins (C/EBP) response elements within Nox1, Nox4 and Nox5 gene promoters. Analysis of Nox1/4/5 gene promoter activities in smooth muscle cells (SMCs) overexpressing C/EBP-α, C/EBP-β or C/EBP-δ (**A**); *n* = 4, ***P < 0.001. *P* values were taken in relation to the empty vector controls. Schematic representation of the Nox gene promoter-luciferase constructs used in co-transfection experiments. The number and the relative positions of the predicted C/EBP sites and the amplicon sizes considered for ChIP assays are depicted (**B**). Chromatin immunoprecipitation analysis of C/EBP-α, C/EBP-β and C/EBP-δ-Nox1/4/5 promoter interaction in IFN-γ-exposed SMCs. Representative agarose gel electrophoresis illustrating the predicted molecular weight of the PCR products (I–V). (**C**). DNA fragments containing- (p21 gene promoter) or lacking- (c-Myc gene promoter) C/EBP elements served as positive or negative controls, respectively (**D**). The symbol (+/−) denotes positive or negative ChIP reactions *n* = 4.

*In silico* analysis (TRANSFAC™) of the human Nox1/4/5 gene promoters indicated the existence of typical C/EBP elements (minimal match of conservation of 90%) that might play a role in the regulation of gene expression. To establish whether the above-mentioned putative binding C/EBP sites promote transcriptional activation of the Nox1/4/5 genes, we performed cotransfection experiments employing specific Nox promoter-luciferase constructs and C/EBP-α, -β and -δ expression vectors. As shown in Figure 6A, transient overexpression of C/EBP-α, -β or -δ up-regulated differentially the luciferase activity directed by the promoters of Nox1, Nox4 and Nox5. Figure 6B shows the schematic representation of the Nox gene promoter-luciferase constructs used in co-transfection experiments, and the predicted number and the relative positions of the C/EBP sites.

To investigate further, the physical interaction among C/EBP proteins and Nox1/4/5 gene promoters, ChIP assay was performed with polyclonal antibodies against C/EBP-α, -β and -δ proteins, and primer sets (≈200 bp amplicon size) spanning the entire promoter (≈1000 bp) region of the Nox1/4/5 genes. Quiescent SMCs were exposed (1 hr) to 50 ng/ml IFN-γ prior to chromatin processing. As depicted by agarose gel (1.5%) electrophoresis, specific C/EBP-α-, -β- and -δ-DNA complexes were detected in various locations of the Nox gene promoters (Fig. 6C). To test the specificity of ChIP assay, various positive and negative controls were used. A DNA fragment derived from the promoter of C/EBP-regulated p21 gene was used as positive control. Negative controls from genomic regions, which do not contain predicted C/EBP elements (*e.g*. c-Myc gene promoter), were employed (Fig. 6D).

## Discussion

Compelling evidence revealed that Nox-derived ROS play a key role in all stages of atherosclerosis and that pharmacological inhibition of Nox function or genetic ablation of various oxidase components protects the vascular cells against the detrimental effects of oxidative stress [7,8,11]. Despite of the numerous existing data, the molecular mechanisms of Nox regulation and the specific function of each Nox subtype remain elusive. In this study, we questioned whether a causal relationship among activated C/EBP transcription factors and Nox activation/expression status exists.

It has been demonstrated that inhibition of C/EBP transcriptional activity by decoy oligodeoxynucleotide reduces restenosis after angioplasty in hypercholesterolaemic rabbits [30]. Moreover, up-regulated Nox-derived ROS are important effectors in restenosis after carotid injury [10]. These data suggest that members of the C/EBP family and Nox play an important role in mediating SMCs proliferation and neointima formation in the carotid arteries.

As up-regulated Nox and C/EBP are associated processes in activated SMCs present within atherosclerotic lesions, we searched for the involvement of C/EBP signalling in the regulation of oxidase expression and function in human aortic SMCs stimulated by IFN-γ, a potent pro-inflammatory cytokine secreted in the atheroma by activated macrophages and artery-infiltrating T cells. It has been demonstrated that IFN-γ acts directly on SMCs to induce cellular hypertrophy and hyperplasia, synthesis of excess extracellular matrix and inflammatory cytokines [31]. Our data confirm and extend these observations. Stimulation of SMCs with increasing doses of IFN-γ led to a significant up-regulation of SMCs impedance-based index. Notably, the changes in cell impedance represent a global measure of various biological activities, including cell attachment, growth, proliferation, migration and matrix deposition, all of which might be severely deregulated in atherosclerosis. In addition, we provide evidence that C/EBP-α play a role in mediating IFN-γ-induced SMCs proliferation.

Our experiments revealed that in human aortic SMCs, IFN-γ enhances Nox activity and intracellular ROS production, a condition that is associated with significant elevations in Nox1, Nox4, and Nox5 gene and protein expression levels. Based on the fact that Nox1, Nox4 and Nox5 isoforms are constitutively expressed in the SMCs and are similarly regulated by IFN-γ, one can predict that their subcellular localization may be an essential condition in determining the function of each Nox subtype [12,32,33]. Moreover, our data bring new evidence that IFN-γ-induced Nox expression is associated with enhanced nuclear translocation of C/EBP-α, -β and -δ transcription factors in response to IFN-γ stimulation.

Multiple mechanisms of C/EBP regulation have been demonstrated in various cell types including phosphorylation, acetylation, activation/repression *via* other transcription factors and autoregulation [34]. However, there are no concluding data for the implication of C/EBP family in the vascular physiology and pathology and their possible impact on Nox expression and function in SMCs.

Studies in different cell types including hepatocytes, adipocytes and gliobastoma cell line demonstrated that C/EBP-β and -δ are activated and up-regulated at transcriptional levels by pro-inflammatory cytokines such as IL-6, IL-1, IFN-γ and TNF-α [19,34,35]. In uninduced states, C/EBP-α and -β isoforms are relatively abundant, while C/EBP-δ mRNA and protein are nearly undetectable in the aforementioned cell types [17,36]. Our data confirm and extend these observations on human aortic SMCs. Short-time stimulation of SMCs (1 hr) with increasing concentrations of IFN-γ led to a significant but dissimilar activation of C/EBP-α, -β and -δ isoforms. Moreover, long time exposure (24 hrs) of SMCs to IFN-γ promoted a gradual increase in the total protein expression levels of the C/EBP-α, -β and -δ subtypes. It is interesting to note that compared with C/EBP-α and -β, the protein expression level of C/EBP-δ was almost undetectable in the total protein extract in unstimulated cells, and was greatly up-regulated following IFN-γ (25–100 ng/ml) treatment. We did, however, detect significant C/EBP-δ protein levels in concentrated nuclear fractions obtained from untreated SMCs. These data suggest the existence of a sequential mechanism of C/EBP activation in response to the severity of inflammatory stress. In agreement with our findings, it has been postulated that C/EBP-δ plays a critical role in the late stage induction of acute phase proteins, while activation transcription factors such as NF-kB and STAT3 are transient and much faster [34].

Interferon-γ activates various members of the C/EBP family *via* mitogen-activated protein kinases (MAPKs)-related signalling pathways, including p38MAPK and ERK1/2, and mixed-linage protein kinases [16,37]. In previous studies, we have shown that pharmacological inhibition of JNK, p38MAPK and ERK1/2 diminished significantly the activated Nox activity and expression in human aortic SMCs exposed to pro-inflammatory conditions [38]. However, the MAPKs control the activity of a large number of transcription factors namely, NF-kB, AP-1 and STAT that are also important regulators of Nox [14,38,39]. Therefore, to overcome this constraint in this study, we used siRNA-mediated gene silencing to down-regulate selectively the expression of various C/EBP isoforms. The results showed that transient silencing of C/EBP-α, -β and -δ reduced significantly but differentially the IFN-γ-induced up-regulation of Nox activity and Nox1-5 mRNA levels. In contrast, a dissimilar regulation was detected at the protein level. Silencing of C/EBP-α, -β or -δ promoted significant decreases in the Nox1 and Nox4 protein levels in IFN-γ-treated cells. Although we have detected significant down-regulations in the Nox5 mRNA expression in cells treated with specific siRNA sequences directed to each of the three C/EBP isoforms, no significant changes in the Nox5 protein level was determined. This evidence suggests that besides C/EBP, alternative signalling pathways, transcription factors and mRNA processing mechanisms may be implicated in the regulation of Nox5 protein in response to IFN-γ stimulation.

Notably, in addition to the major catalytic components (*e.g*. Nox1, Nox4 and Nox5), the potential regulation of other proteins comprising the Nox complex, up-stream regulators of Nox, as well as expression-dependent and expression-independent mechanisms affecting Nox activity, should be considered.

To further investigate the implication of C/EBP family in the regulation of Nox expression, *in silico* analysis (TRANSFAC™) of the promoters of human Nox1, Nox4 and Nox5 genes was done to predict the occurrence of C/EBP elements. The program identified the existence of typical C/EBP sites in the promoters of Nox1, Nox4 and Nox5 that might play a role in the regulation of gene expression.

To gain additional mechanistically insights into the molecular basis of Nox regulation by C/EBP-α, -β and -δ, various promoter analyses were done. Transient overexpression of each of C/EBP-α, -β or -δ induced significant increases in the luciferase level directed by promoters of human Nox1/4/5 genes. These data suggest that C/EBP could regulate the transcription of Nox genes by either direct or indirect mechanisms. Furthermore, specific C/EBP-α, -β and -δ– Nox1/4/5 promoters interactions were identified in various locations by ChIP assays.

Activated C/EBP transcription factors bind specific elements RTTGCGYAAY (R: A/G, Y: C/T) in the enhancer or promoter regions to control the transcription of target genes. Nevertheless, specific interactions with less conserved DNA-binding sites have been shown [40]. Besides canonical C/EBP–promoter interactions, a number of alternative mechanisms of C/EBP-mediated gene expression have been proposed including interplays with other transcription factors (NF-kB, AP-1, Sp1, CREB) [41–43] or recruitment of various regulatory proteins that modify the chromatin conformation, a condition that facilitates the trapping of basal transcription factors [44,45]. Considering the fact that NF-kB, AP-1, STAT1/3 and Sp1 are direct regulators of various Nox isoforms [46], the existence of diverse associations among transcriptional complexes formed by heterodimers consisting of C/EBP and other transcription factors should be considered as well.

Collectively, the reported data indicate the existence of C/EBP-dependent mechanisms involved in the regulation of Nox in human aortic SMCs exposed to pro-inflammatory conditions. Based on the fact that IFN-γ also stimulates C/EBP expression, activation of these transcription factors may contribute to a sustained induction of Nox in cardiovascular disorders. In addition, a similar pattern of Nox regulation was detected in AngII-treated SMCs suggesting that C/EBP transcription factors may transduce the signals of other physiological and pathological stimuli that control Nox expression and function.

To our knowledge, this is the first report providing evidence on the regulation of Nox by C/EBP family in vascular cells. As C/EBP transcription factors transduce the signals of numerous pro-atherogenic stimuli, pharmacological targeting of C/EBP activity and/or expression could be a novel strategy to counteract oxidative stress by modulating Nox and to improve vascular functions in vascular inflammatory disorders such as atherosclerosis.

## Supporting information

**Figure S1.** Implication of C/EBP transcription factors in mediating SMCs proliferation. **Figure S2.** AngII induces Nox activity *via* C/EBP-mediated mechanisms. **Table S1.** Sequences of oligonucleotide primers used in the ChIP assays to amplify various regions of Nox1, Nox4 and Nox5 gene promoters.
