# Supplementary material for: C/EBP transcription factors regulate NADPH oxidase in human aortic smooth muscle cells
Source: J Cell Mol Med. 2014 May 6;18(7):1467–77. doi: 10.1111/jcmm.12289 (PMC4124029; doi:10.1111/jcmm.12289)
Supplement: Supplementary file 1 [file jcmm0018-1467-SD1.doc]

**Supporting information**

**C/EBP transcription factors regulate NADPH oxidase in human aortic smooth muscle cells**

Simona-Adriana Manea, Andra Todirita, Monica Raicu, Adrian Manea*****

Institute of Cellular Biology and Pathology “Nicolae Simionescu” of the Romanian Academy, Bucharest, Romania

**^*^Corresponding author:**

**Adrian Manea, Ph.D.**

**e-mail:** [adrian.manea@icbp.ro](mailto:adrian.manea@icbp.ro)

**Institute of Cellular Biology and Pathology “Nicolae Simionescu”,**

**Molecular and Cellular Pharmacology - Functional Genomics Laboratory**

**8, B.P. Hasdeu Street, 050568, Bucharest, Romania**

**Tel: (+4021) 319 27 37**

**Fax: (+4021) 319 45 19**

**
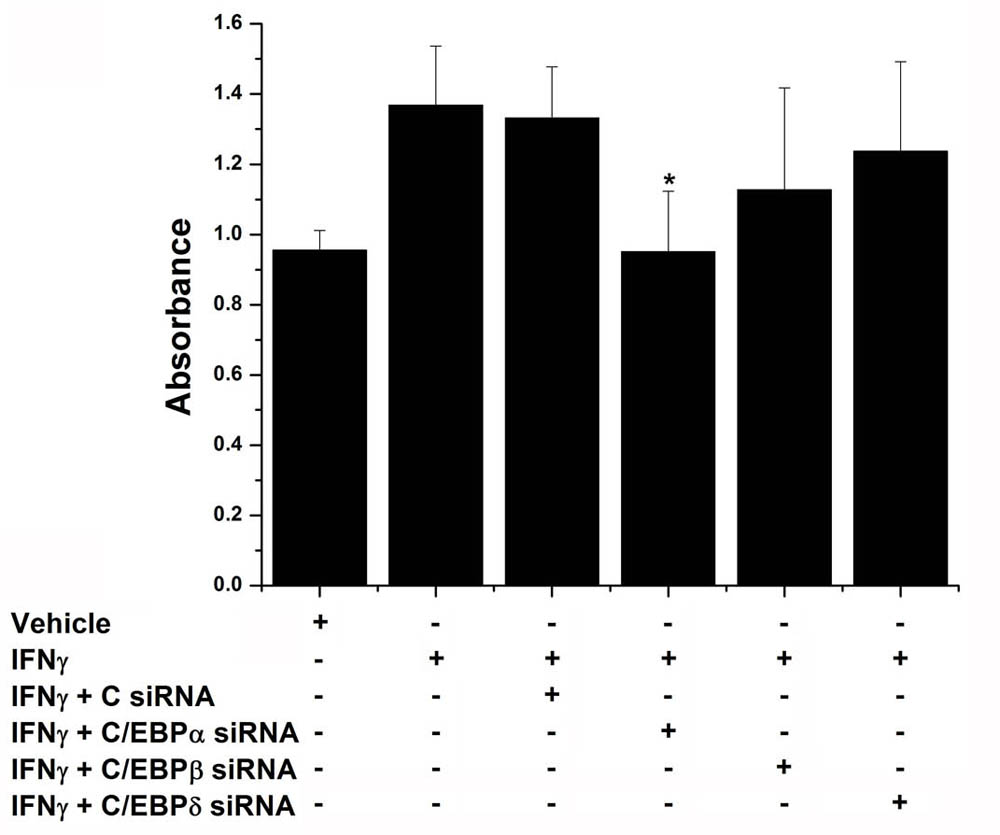
**

**Figure S1.** Implication of C/EBP transcription factors in mediating SMCs proliferation. Quiescent SMCs were exposed (24 h) to 50 ng/ml IFNγ in the absence/presence of siRNA sequences directed to silence the expression of various C/EBP subtypes. Cell proliferation was assessed by MTT assay. n=4, **P*<0.05. *P*-values were taken in relation to C siRNA-transfected cells stimulated with IFNγ.

**
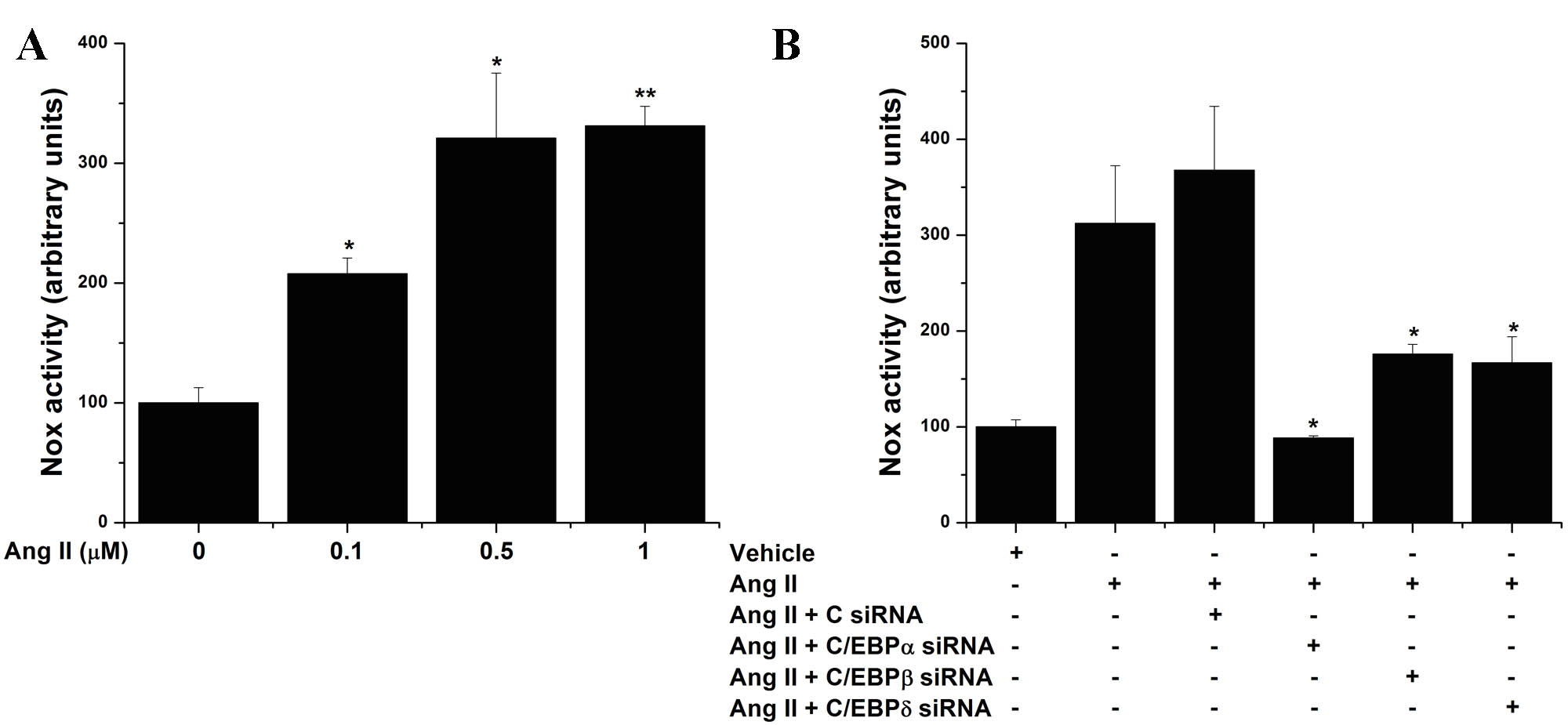
**

**Figure S2.** AngII induces Nox activity via C/EBP-mediated mechanisms. Dose-dependent effect of AngII on Nox activity in SMCs exposed to AngII (0.1-1 μM) for 24 h **(A)**. n=3, **P*<0.05, ***P*<0.01. *P*-values were taken in relation to vehicle-exposed cells. Quiescent SMCs were exposed (24 h) to 1 μM AngII in the absence/presence of siRNA sequences directed to silence the expression of various C/EBP subtypes. Nox activity was determined by lucigenin-enhanced chemiluminescence **(B)**. n=3, **P*<0.05. *P*-values were taken in relation to C siRNA-transfected cells stimulated with AngII.

**
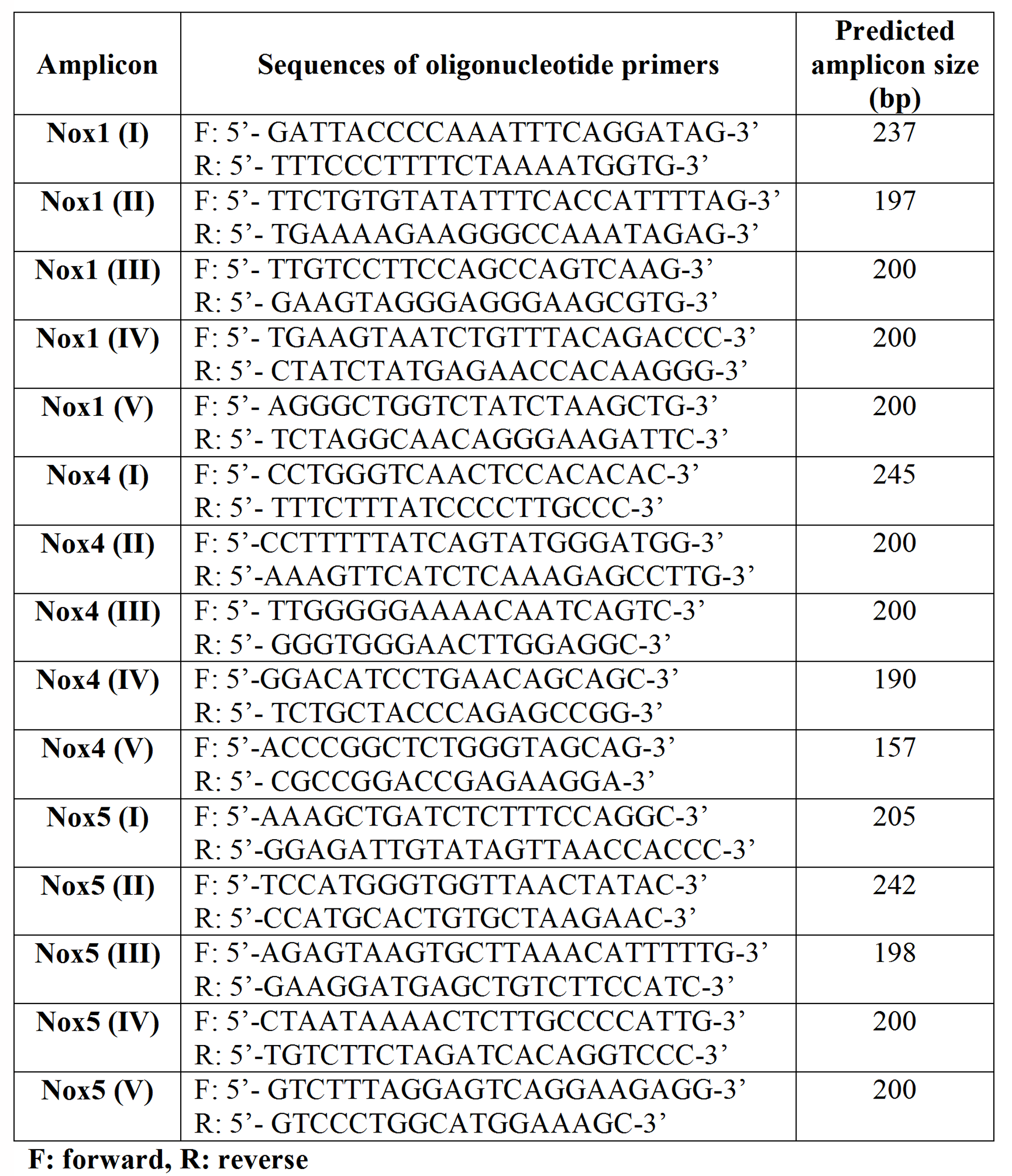
**

**Table S1.** Sequences of oligonucleotide primers used in the ChIP assays to amplify various regions of Nox1, Nox4, and Nox5 gene promoters.
